# Distinct Subgroups of Patients With Lung Cancer Receiving Chemotherapy: A Latent Transition Analysis

**DOI:** 10.3389/fonc.2020.522407

**Published:** 2020-10-14

**Authors:** Nannan Li, Lili Hou, Shu Li

**Affiliations:** ^1^School of Nursing, Shanghai Jiao Tong University, Shanghai, China; ^2^Department of Nursing, Shanghai Ninth People’s Hospital, Shanghai Jiao Tong University School of Medicine, Shanghai, China; ^3^Department of Nursing, Shanghai Seventh People’s Hospital, Shanghai, China

**Keywords:** lung cancer patients, symptom experience, chemotherapy, latent transition analysis, symptom profile

## Abstract

**Objectives:**

To identify subgroups of patients with lung cancer receiving chemotherapy based on the severity dimension of symptom experience, and to examine changes in membership between these subgroups over time.

**Methods:**

Patients who were scheduled to receive chemotherapy completed the Chinese version of the MD Anderson Symptom Inventory and the revised lung cancer module with a total of 19 symptom items. Data were collected at three time points: two weeks before chemotherapy (T1), after chemotherapy cycle 1 (T2), and after chemotherapy cycle 3 or above (T3). The latent profile analysis and latent transition analysis were used to identify underlying subgroups and describe changes in subgroup membership over time.

**Results:**

From the total sample (*N* = 195), 160 patients completed the symptom assessment at T1, T2, and T3. Two distinct latent symptom profiles of patients could be identified at T1, T2, and T3, which were classified as “Mild” and “Moderate-Severe” profiles. From T1 to T2 and T3, members in the Mild profile were more likely to move to the Moderate-Severe profile. Chemotherapy protocols, prior surgery treatment, and level of education can predict the transitions.

**Conclusion:**

Results provide a better understanding of the patient’s different symptom experiences and characteristics. These could help clinicians to anticipate symptom patterns and develop interventions in lung cancer patients who were scheduled to receive chemotherapy for the first time.

## Introduction

Individuals with lung cancer experience multiple symptoms, which are frequently associated with disease and side effects of treatment (1). A number of physical and psychological symptoms were reported during the chemotherapy trajectory (2, 3). An early study has shown that symptoms are related to each other and appear simultaneously, and this phenomenon is described as “symptom cluster” (4). Compared with single symptoms, clusters may have more deleterious influences on patient outcomes, such as quality of life, functional status, and survival (5). Identifying symptom clusters with validity is paramount for developing targeted interventions aimed at improving clinical outcomes of patients.

Two conceptual approaches to symptom cluster analysis have been demonstrated in the current research. The first common approach was categorizing symptoms, which is a “variable-oriented” approach that focused on symptom variables (6). Most common statistical analysis, such as principal components analysis (PCA) (7), factor analysis (FA) (8), and cluster analysis (9) were used to identify symptom clusters in patients with lung cancer. Emotional or psychological and gastrointestinal symptom clusters were commonly identified. However, individual variability may not have been considered in prior studies. In addition, symptom cluster patterns may change over time (10). The statistical methods mentioned above are advantages in dealing with the cross-sectional data, but for some longitudinal data, they are limited to tracking individual trajectories of symptom experiences over the course of a disease or treatment.

Another approach focused on being “person-oriented” and grouped patients by their symptom patterns. Latent class analysis (LCA) can provide a model-based method to grouping patients using categorical variables (11, 12), and members in each latent class have similar symptom experiences. Latent class analysis is called latent profile analysis (LPA) when using continuous variables (12). Latent transition analysis (LTA) is an extension of LCA that identifies changes in latent class membership using longitudinal data. It can estimate the transition probability of individuals who move from one latent class to another at different time points (11, 12). These analytical methods have received growing attention in cancer symptom cluster research over recent years. A few cross-sectional studies (13–15) classified patients into subgroups using LCA, but a limited number of studies used longitudinal data. Four studies explored subgroups of patients prior and after chemotherapy based on symptom occurrence or severity in a heterogeneous sample (16, 17) or specific cancer type of patients (18, 19). Different distinct subgroups, transition status, and patient characteristics were identified using LCA and LTA.

Previous studies have provided a deep understanding of grouping patients and their changes over time. To our knowledge, there has been no research conducted in patients with lung cancer. To better understand the interindividual variability among patients with lung cancer, this study aims to identify the latent profiles based on the severity dimension of symptom experience, to compare the characteristics of different group patients, and to examine the changes in subgroup membership over time.

## Materials and Methods

### Patient Selection and Study Design

A total of 195 participants were recruited using convenience sampling. Inclusion criteria included: being older than 18 years; able to read and speak Chinese; a diagnosis of lung cancer; scheduled to receive chemotherapy for the first time; chemotherapy protocols were platinum-containing a two-drug combination; and without cognitive impairment.

This study was a prospective, longitudinal study. Data were collected at three time points: two weeks before chemotherapy (T1), within one week after chemotherapy cycle 1, and after cycle 3 or above (T2 and T3). This study was approved by the hospital ethics committee.

### Measures

#### Demographic and Medical Characteristics

Demographic variables included age, gender, educational level, and income. Medical characteristics included prior treatment, comorbidities, cancer stage, and chemotherapy protocols.

#### Chinese Version of the MD Anderson Symptom Inventory

The MD Anderson Symptom Inventory (MDASI) was used to evaluate the severity of symptoms and symptom interference with daily life (20). It consists of 13 core symptom items and six symptom interference items. Each item was measured using a 0 (not present) to 10 (as bad as can imagine) numeric rating scale. The MDASI has been translated into a Chinese version (MDASI-C), which has good internal consistency (Cronbach’s α ranges from 0.84 to 0.90) (21). For this study, the 13 symptom items of the MDASI-C were used.

#### Revised Lung Cancer Module of the MDASI

The lung cancer module of the MDASI was developed from the MDASI, which consists of cough, constipation, and sore throat (22). Researchers revised this module to measure the symptom burden of Chinese patients with lung cancer (23). The revised version of the lung cancer module consists of cough, expectoration, hemoptysis, chest tightness, constipation, and weight loss. It has an acceptable internal consistency reliability (0.773) and content validity (0.944) (23).

### Statistical Analysis

Data were analyzed using Mplus 7.4 and SPSS version 21.0. The LPA and LTA were conducted to identify subgroups of patients with lung cancer and explore transitions in subgroup membership over time, using the symptom severity data. To have a sufficient number of patients with each symptom in LPA and LTA, symptoms that occurred in less than 40% of patients at three time points were excluded from the analysis, and were not used in these studies (17, 24).

First, separate LPAs were performed to identify latent profiles of patients with distinct symptom patterns at three time points. The number of latent profiles for each LPA was determined by model comparison. Several statistical fit indices, including the Akaike Information Criteria (AIC), Bayesian Information Criteria (BIC) values, and the sample-size-adjusted BIC (Adj.BIC), were applied to compare the models, with smaller BIC, AIC, and Adj.BIC indicating a better-fitting model (25). Lo–Mendell–Rubin (LMR) adjusted likelihood ratio and the bootstrapped likelihood ratio test (BLRT) were used to compare the fit of the k and the k − 1 profile model. The significant *p* value (*p* < 0.05) in these tests would indicate that the k profile model fits data better than the k − 1 profile model (11). The entropy value indicates the profile classification accuracy, and the higher the entropy the better the model is.

After identifying the optimal latent profile model at each time point, we extended the LPA models to LTA to examine the transitions in membership between latent profiles over time. The log-likelihood was used for model comparison and confirmation. To ensure that the maximum likelihood solution can be identified for LTA models, 100 sets of random starting values were used to examine the percentage of potential solutions that converged to the maximum likelihood solution. A higher percentage represents the model was sufficiently well identified (26). In LTA, the possible transitions of latent profiles from T1 → T2 → T3 were estimated. The latent profile membership and transitions were saved as “observed” categorical variables for further analysis. Descriptive statistics in each profile, such as patients’ demographics, medical characteristics, and symptom severity, were analyzed and compared using SPSS version 21.0. Logistic regression was used to examine if participant characteristics predict the latent profile transitions.

Before LTA, we tested a longitudinal measurement invariance across time. Measurement invariance assumes that all the measurement parameters are equal across all the time points and the meanings of profiles are the same at each time point, thus the model can be interpreted meaningfully (27). A fixed sequence of model comparison was conducted, which involved configural invariance, weak invariance, strong invariance, strict invariance, invariant factor variances, and invariant factor covariances models with restrictive constraints imposed (28). The chi-square difference test and a change (Δ) in CFI (comparative fit index) were used to compare those six nested models. A non-significant chi-square difference test indicates the current model was more suitable than the previous one; a value of ΔCFI smaller than −0.01 indicates that the hypothesis of invariance should not be rejected. The change in CFI are superior to chi-square difference tests due to their stability (29).

## Results

### Latent Profile Analyses

In total, 160 patients completed the symptom assessment at T1, T2, and T3. A total of four out of the 19 symptoms (pain, numbness, hemoptysis, and weight loss) that occurred in less than 40% at all three time points were excluded in the LPA. Separate LPAs were performed at each time point. Supplementary Table 1 provides the fit statistics for models ranging from one to five latent profiles. At T1, the two-profile model had lower AIC, BIC, and Adj.BIC values than the one-profile model, and the LMR and BLRT statistics were significant for the two-profile model, indicating that the two-profile model fits data better than the one-profile model. Although the three-, four-, and five-profile models had lower AIC, BIC, and Adj.BIC values in comparison to the two-profile model, the LMR statistics were not significant. Thus, the two-profile model fits data well. At T2 and T3, similar LPA models were found. The two-profile model was selected as the best fit model at each time point. The entropy value was 0.98, 0.913, and 0.937 at T1, T2, and T3, respectively.

### Longitudinal Measurement Invariance

The results of longitudinal measurement invariance across time are presented in Supplementary Table 2. Chi-square difference test showed that model 2 is significantly different from model 1, but the change in CFI (ΔCFI < 0.01) supported model 2’s constraining equal factor loadings. Other results were based on non-significant chi-square difference tests (*p* > 0.05) and the change in CFI (ΔCFI < 0.01) between models, suggesting that there is evidence of strong, strict, factor variances, and factor covariances being invariant. Thus, equal restrictions were imposed on measurement parameters across time.

### Latent Transition Analysis

Latent transition analysis was done using longitudinal data from three time points of measurement. Models with different numbers of latent profiles were compared (Supplementary Table 3). Results showed that models with four or more profiles were unidentified because of not converging. The two-profile model had a higher percentage of solutions that converged to the maximum likelihood solution. Additionally, combined with the results of LPAs determined above, we selected the two-profile model (entropy value = 0.953) as the best fit model. According to the mean score of symptom severity at each time point, profile 1 was classified as “Mild,” and profile 2 was classified as “Moderate-Severe.” Supplementary Figure 1 shows the mean scores of multiple symptoms by profile from the LTA model.

### Differences in Sample Characteristics Among the Two Profiles

Supplementary Table 4 presents the differences in demographic and medical characteristics. At T1, there were significant differences in gender between the Mild and Moderate-Severe profiles. Patients in the Moderate-Severe profile were more likely to be female.

### Transitions Between Latent Profiles and Predictors

In LTA, the transition probabilities of latent profiles between three time points were estimated (Supplementary Table 5). Members in the Mild profile at T1 were more likely to transition. About 59.1% of them remained at T2, while 40.9% transitioned to the Moderate-Severe profile. Members in the Moderate-Severe profile at T1 were relatively stable; only two patients transitioned to the Mild profile at T2. From T2 to T3, members in two profiles were both relatively stable, with 88.8 and 95.5% of them remaining in the Mild and Moderate-Severe profile. Only 11.2% in the Mild profile moved to the Moderate-Severe profile. 0.5% in the Moderate-Severe profile moved to the Mild profile. From T1 to T3, 53.9% of patients in the Mild profile remained at T3, while 46.1% transitioned to the Moderate-Severe profile. Among patients in the Moderate-Severe profile at T1, 75% of them remained at T3, while 25% transitioned to the Mild profile.

Given the two latent profiles at each time point, there are eight transition patterns from T1 → T2 → T3. As shown in Supplementary Table 5, pattern 1 (patients in the Mild profile at T1 remained over time) and pattern 4 (patients in the Mild profile at T1 transitioned to the Moderate-Severe profile at T2 and T3) were the most common transition patterns, accounting for 86.88% of all transitions. Logistic regression revealed that the chemotherapy protocol can predict pattern 1; prior surgery treatment and level of education can predict pattern 4 (see Supplementary Table 6).

## Discussion

This study is the first to identify subgroups of patients with lung cancer using LPA and LTA. We identified two distinct subgroups of patients based on the severity of 15 symptoms from prior to and following chemotherapy. Patient characteristics among subgroups and the transitions in patients over time were reported. These results provided evidence for the person-oriented method on symptom cluster research.

### Two Latent Profiles at Each of the Three Time Points

In this population, two latent symptom profiles could be identified at T1, T2, and T3. Profile 1 was classified as “Mild” and had a relatively low symptom score. Profile 2 was classified as “Moderate-Severe” and had a moderate to severe score for multiple symptoms. This classification based on symptom scores is consistent with symptom control practice guidelines (30, 31). Similar grouping results were found in children undergoing chemotherapy, which were defined as “less severe symptoms” and “severe symptoms” (18). A previous LTA study (17) identified three latent classes (low, moderate, high) using symptom occurrence in oncology (including lung cancer) outpatients, which differs from our findings. This is possibly due to the small sample size, or the different symptom dimension used.

### Differences Patient Characteristics Among Two Profiles

There were significant differences in gender among the two profiles before chemotherapy. Patients in the Moderate-Severe profile were more likely to be female. This finding was similar to a prior study (32) which found that females experienced higher levels of physical and psychological symptoms. Although there are relatively fewer females than males in the lung cancer population, more attention and support should be given them.

### Transitions Between Latent Profiles and Predictors

In our study, the most common transition between latent profiles occurred in the Mild profile at T1. Among 152 patients, about half of them remained over time, and patients who were receiving the GP protocol were more likely to remain in the Mild profile than those receiving AP protocol. However, the toxic reaction of AP and GP protocols revealed no difference in patients with non-small-cell lung cancer (33). This is mainly because most patients were receiving the AP protocol in this study. Half transitioned to the Moderate-Severe profile after chemotherapy cycles. This finding suggests that some patients experienced a worsening symptom burden after chemotherapy cycle 1, and these symptom experiences may persist during chemotherapy. As reported by Wang et al., this phenomenon may be a predictor of overall survival (34).

Our study showed that patients who had surgery treatment and had low education levels before chemotherapy were more likely to transition to the Moderate-Severe profile over the course of chemotherapy cycles than patients who had no surgery treatment and had high education levels. These predictors were also reported in previous results (32, 35). Thus, symptom support should be given to these patients. Additionally, membership in the two profiles were both relatively stable during chemotherapy cycles. This result has not been reported in patients with lung cancer, but was consistent with the previous report that used LTA to examine the changes in profile status among children patients, which revealed that subgroup membership remains relatively stable from the start to the mid-way cycle of chemotherapy (18).

### Implications for Practice

This study focuses on patients receiving chemotherapy for the first time, characterizing patients into “Mild” and “Moderate-Severe” subgroups based on multiple symptom experience. Importantly, patient characteristics between groups were observed and were shown to have great significance for clinical practice. The tailored severity-based symptom intervention strategies can be developed for specific populations. According to our findings, the majority of transitions were found in the “Mild” group, in which patients moved to the “Moderate-Severe” group. This provides a better understanding toward the change in symptom experience over the course of chemotherapy. The predictive factors of transitions may help clinicians to pay more attention to patients who had surgery treatment and had low education levels when implementing interventions. It is important to promote positive transitions in lung cancer patients receiving chemotherapy.

## Limitations and Conclusion

Several limitations should be considered. The relatively small sample size may have limited the number of distinct categories grouping patients. A small group (*N* = 8) of patients were identified prior to chemotherapy, thus, this classification needs to be further verified. Additionally, we did not evaluate symptoms through the consecutive cycles and after completion of chemotherapy. Finally, four symptoms with occurrence rates of less than 40% were excluded in LPA and LTA, and the effects of these symptoms may be ignored.

In conclusion, we identified the Mild and Moderate-Severe subgroups in patients with lung cancer prior to and following their cycles of chemotherapy. Two distinct symptom patterns were observed in symptom scores. Patients in the Mild profile were more likely to move to the Moderate-Severe profile after the cycles of chemotherapy. These findings could help clinicians to anticipate symptom patterns and develop interventions in lung cancer patients who were scheduled to receive chemotherapy for the first time.

## Data Availability Statement

The datasets generated for this study are available on request to the corresponding author.

## Ethics Statement

The studies involving human participants were reviewed and approved by The ethics committee of Shanghai Seventh People’s Hospital. The patients/participants provided their written informed consent to participate in this study.

## Author Contributions

LH provided guidance for the design of the study and revised the manuscript. NL and SL collected the data. NL analyzed the data and wrote the manuscript. All authors contributed significantly and were in agreement with the content of the manuscript.

## Conflict of Interest

The authors declare that the research was conducted in the absence of any commercial or financial relationships that could be construed as a potential conflict of interest.
