# Supplementary material for: Distinct Subgroups of Patients With Lung Cancer Receiving Chemotherapy: A Latent Transition Analysis
Source: Front Oncol. 2020 Oct 14;10:522407. doi: 10.3389/fonc.2020.522407 (PMC7591394; doi:10.3389/fonc.2020.522407)
Supplement: Supplementary file 1 [file Data_Sheet_1.docx]

Supplementary Material

# Supplementary Figures and Tables

## Supplementary Figures

**Figure legend**

**Figure1.** **Mean scores of multiple symptoms by profile from the LTA model**

Each broken line represents the mean score of symptom severity at each time point grouped by patients. T1: two weeks before chemotherapy; T2: cycle 1 of chemotherapy; T3: cycle 3 or above of chemotherapy

## Supplementary Tables

**Table 1 Results of LPA at each time point**

|  | LL | FP | AIC | BIC | Adj.BIC | LMR (p) | BLRT (p) | Entropy |
| --- | --- | --- | --- | --- | --- | --- | --- | --- |
| T1 |  |  |  |  |  |  |  |  |
| 1-profile | -5379.21 | 30 | 10818.43 | 10910.68 | 10815.71 | - | - | - |
| 2-profile | -5112.57 | 46 | 10317.14 | 10458.60 | 10312.98 | 0.0009 | 0.0000 | 0.980 |
| 3-profile | -4937.79 | 62 | 9999.58 | 10190.24 | 9993.97 | 0.2579 | 0.0000 | 0.988 |
| 4-profile | -4867.06 | 78 | 9890.11 | 10129.98 | 9883.06 | 0.7497 | 0.0000 | 0.975 |
| 5-profile | -4807.30 | 94 | 9802.60 | 10091.66 | 9794.09 | 0.6487 | 0.0000 | 0.971 |
| T2 |  |  |  |  |  |  |  |  |
| 1-profile | -5719.29 | 30 | 11498.59 | 11590.84 | 11495.87 | - | - | - |
| 2-profile | -5442.93 | 46 | 10977.85 | 11119.31 | 10973.69 | 0.0011 | 0.0000 | 0.913 |
| 3-profile | -5347.93 | 62 | 10819.86 | 11010.52 | 10814.26 | 0.2131 | 0.0000 | 0.917 |
| 4-profile | -5270.52 | 78 | 10697.05 | 10936.91 | 10689.99 | 0.5707 | 0.0000 | 0.932 |
| 5-profile | -5208.50 | 94 | 10605.00 | 10894.07 | 10596.50 | 0.3562 | 0.0000 | 0.947 |
| T3 |  |  |  |  |  |  |  |  |
| 1-profile | -5751.47 | 30 | 11562.95 | 11655.20 | 11560.23 | - | - | - |
| 2-profile | -5464.10 | 46 | 11020.19 | 11161.65 | 11016.03 | 0.0001 | 0.0000 | 0.937 |
| 3-profile | -5385.84 | 62 | 10895.68 | 11086.34 | 10890.08 | 0.3772 | 0.0000 | 0.908 |
| 4-profile | -5314.40 | 78 | 10784.81 | 11024.67 | 10777.75 | 0.2031 | 0.0000 | 0.938 |
| 5-profile | -5258.01 | 94 | 10704.01 | 10993.08 | 10695.51 | 0.5019 | 0.0000 | 0.953 |

LL, log-likelihood; FP, free parameters; AIC, Akaike information criteria; BIC, Bayesian information criteria; Adj.BIC: sample-size-adjusted BIC; LMR, Lo-Mendell-Rubin Adjusted Likelihood Ratio test. BLRT, bootstrapped likelihood ratio test.

**Table 2 Longitudinal measurement invariance across time**

| Model | χ^2^ | df | CFI | Model comparisons | Δχ^2^ | Δdf | *p* | ΔCFI |
| --- | --- | --- | --- | --- | --- | --- | --- | --- |
| Model 1 | 1319.84 | 834 | 0.902 | - | - | - | - |  |
| Model 2 | 1370.17 | 856 | 0.897 | Model 2 vs Model 1 | 50.33 | 22 | 0.000 | -0.005 |
| Model 3 | 1388.96 | 867 | 0.895 | Model 3 vs Model 2 | 18.79 | 11 | 0.065 | -0.002 |
| Model 4 | 1403.15 | 882 | 0.895 | Model 4 vs Model 3 | 14.19 | 15 | 0.511 | 0.000 |
| Model 5 | 1405.05 | 886 | 0.896 | Model 5 vs Model 4 | 1.9 | 4 | 0.754 | 0.001 |
| Model 6 | 1416.65 | 892 | 0.895 | Model 6 vs Model 5 | 11.6 | 6 | 0.072 | -0.001 |

df, degrees of freedom; CFI, comparative fit index; Model 1, configural invariance, no parameters constrained to be equal across time; Model 2, weak invariance, factor loadings are constrained to be equal across time; Model 3, strong invariance, weak invariance plus item intercept are constrained to be equal across time; Model 4, strict invariance, strong invariance plus equal error variance; Model 5, factor variances invariance, strict invariance plus equal factor variances; Model 6, factor covariances invariance, factor variances invariance plus equal factor covariances.

**Table 3 Model fit statistics for LTA**

| Model | LL | FP | AIC | BIC | Entropy | % |
| --- | --- | --- | --- | --- | --- | --- |
| 2-profile | -16174.60 | 80 | 32509.19 | 32755.20 | 0.953 | 40% |
| 3-profile | -15863.29 | 104 | 31934.58 | 32254.40 | 0.961 | 32% |

LL, log-likelihood; FP, free parameters; AIC, Akaike information criteria; BIC, Bayesian information criteria; %, percentage of the solution converging to the maximum likelihood.

**Table 4 Differences in Demographic and Clinical Characteristics among the two profiles**

| Characteristics | Time1 N(%) | | Statistics |
| --- | --- | --- | --- |
|  | Profile 1 (n=152) | Profile 2 (n=8) |  |
| **Age** | 62.06±8.58 | 59.13±10.16 | 0.87 |
| **Gender** |  |  |  |
| Male | 114(75.0) | 0(0) | 21.02^*^ |
| Female | 38(25.0) | 8(100%) |  |
| **Education** |  |  |  |
| Elementary | 35(23.0) | 2(25.0) | -0.03 |
| Junior high | 55(36.2) | 3(37.5) |  |
| Senior high | 33(21.7) | 1(12.5) |  |
| University | 29(19.1) | 2(25.0) |  |
| **Income** |  |  |  |
| ＜2000 | 39(25.7) | 1(12.5) | -0.09 |
| 2000-3000 | 34(22.4) | 3(37.5) |  |
| 3000-5000 | 50(32.9) | 3(37.5) |  |
| ＞5000 | 29(19.1) | 1(12.5) |  |
| **Prior treatment** |  |  |  |
| **Surgery** |  |  |  |
| Yes | 33(21.7) | 3(37.5) | 0.37 |
| No | 119(78.3) | 5(62.5) |  |
| **Radiotherapy** |  |  |  |
| Yes | 9(5.9) | 2(25.0) | 1.86 |
| No | 143(94.1) | 6(75.0) |  |
| **Comorbidities** |  |  |  |
| Yes | 70(46.1) | 5(62.5) | 0.30 |
| No | 82(53.9) | 3(37.5) |  |
| **Stage** |  |  |  |
| I | 5(3.3) | 0(0) | -1.06 |
| II | 6(3.9) | 1 (12.5) |  |
| Ⅲ | 63(41.4) | 1 (12.5) |  |
| IV | 78(51.3) | 6 (75.0) |  |

^*^ *p*＜0.05

**Table 5** **Profile Transition probabilities over time**

|  | | **Transition from T1(Rows) to T2(Columns)** | | | | |
| --- | --- | --- | --- | --- | --- | --- |
|  |  | Profile 1 (N=91) | | Profile 2 (N=69) | | |
| Profile 1 (N=152) | | 0.591 | | 0.409 | | |
| Profile 2 (N=8) | | 0.250 | | 0.750 | | |
|  | | **Transition from T2(Rows) to T3(Columns)** | | | | |
|  | | Profile 1 (N=84) | | Profile 2 (N=76) | | |
| Profile 1 (N=91) | | 0.888 | | 0.112 | | |
| Profile 2 (N=69) | | 0.045 | | 0.955 | | |
|  | | **Transition from T1(Rows) to T3(Columns)** | | | | |
|  | | Profile 1 (N=84) | | Profile 2 (N=76) | | |
| Profile 1 (N=152) | | 0.539 | | 0.461 | | |
| Profile 2 (N=8) | | 0.250 | | 0.750 | | |
| **Transition Patterns from**  **T1→T2→T3** | | | | | | |
|  | Pattern | | T1→T2→T3 | | N (%) | Transition Probabilities |
| Profile 1  N=152 | Pattern 1 | | Profile 1**→**Profile 1**→**Profile 1 | | 79 (49.38) | 0.520 |
|  | Pattern 2 | | Profile 1**→**Profile 1**→**Profile 2 | | 10 (6.25) | 0.066 |
|  | Pattern 3 | | Profile 1**→**Profile 2**→**Profile 1 | | 3 (1.88) | 0.020 |
|  | Pattern 4 | | Profile 1**→**Profile 2**→**Profile 2 | | 60 (37.5) | 0.395 |
| Profile 2  N=8 | Pattern 5 | | Profile 2**→**Profile 1**→**Profile 1 | | 2 (1.25) | 0.250 |
|  | Pattern 6 | | Profile 2**→**Profile 1**→**Profile 2 | | 0 (0.00) | 0.000 |
|  | Pattern 7 | | Profile 2**→**Profile 2**→**Profile 1 | | 0 (0.00) | 0.000 |
|  | Pattern 8 | | Profile 2**→**Profile 2**→**Profile 2 | | 6 (3.75) | 0.750 |

**Table 6**

**Odds ratios of each covariate predicting the selected transitions**

| Covariate | OR (% C.I.) | |
| --- | --- | --- |
|  | Pattern 1 | Pattern 4 |
| Age | 1.00 (0.95,1.05) | 0.98 (0.93,1.03) |
| Gender (ref. male) | 0.45 (0.16,1.28) | 1.49 (0.55,4.04) |
| Prior treatment |  |  |
| Surgery (ref. no) | 0.21 (0.08,0.56) | 3.73 (1.44,9.64)^*^ |
| Radiotherapy (ref. no) | 0.24 (0.03,1.78) | 5.07 (0.71,36.13) |
| Comorbidities (ref. no) | 0.73 (0.30,1.75) | 1.19 (0.49,2.92) |
| Education (ref. University) |  |  |
| Elementary | 0.21 (0.06,0.71) | 0.02 (4.55,1.34)^*^ |
| Junior high | 1.41 (0.49,4.04) | 0.41 (0.65,0.23) |
| Senior high | 1.82 (0.56,5.99) | 0.12 (0.38,0.11) |
| Income (ref.＜2000) |  |  |
| 2000-3000 | 0.80 (0.23,2.75) | 1.33 (0.37,4.78) |
| 3000-5000 | 1.44 (0.45,4.59) | 1.02 (0.32,3.32) |
| ＞5000 | 1.06 (0.22,5.05) | 1.76 (0.35,8.86) |
| Stage (ref. I) |  |  |
| II | 0.11 (0.00,2.92) | 0.85 (0.07,11.24) |
| Ⅲ | 0.27 (0.02,3.97) | 0.62 (0.04,9.22) |
| IV | 0.29 (0.02,3.53) | 1.44 (0.12,17.48) |
| Chemotherapy protocol (ref.AP) |  |  |
| DP | 3.55 (0.59,21.52) | 0.59 (0.10,3.47) |
| GP | 3.06 (1.17,8.00)^*^ | 0.46 (0.18,1.19) |
| EP | 5.90 (1.98,17.60) | 0.14 (0.04,0.47) |
| NP/TP | 2.85 (0.84,9.66) | 0.61 (0.18,2.05) |

^*^ *p*＜0.05; ref, reference variable.
